# Supplementary material for: MiR-223-3p in Cancer Development and Cancer Drug Resistance: Same Coin, Different Faces
Source: Int J Mol Sci. 2024 Jul 26;25(15):8191. doi: 10.3390/ijms25158191 (PMC11311375; doi:10.3390/ijms25158191)
Supplement: Supplementary file 1 [file ijms-25-08191-s001.zip › Figure S1.pdf]

CRC	2	8
NSCLC	5	3
BC	6	2
OC	0	3
PCa	1	1
LGG / GBM	5	1
PC	0	3
AML	3	1
CML	2	0
ALL	1	4
CLL	1	0

Figure S1. Heatmap showing the number of articles cited in this review that defines miR-223 as oncosuppressive (blues) or oncogenic (yellows) miRNA, for each type of cancer reviewed. The colour intensity of the heatmap is directly proportional to the number of references supporting the specific function of the miRNA: the less is the number of references citing the oncosuppressive or oncogenic role the lighter is the blue or the yellow, respectively; the more is the the number of references citing the oncosuppressive or oncogenic role the deeper is the blue or the yellow. The number of references supporting the function of miR-223 is reported as arabic numbers within the heatmap, for each tumor type. CRC: colorectal carcinoma; NSCLC: non-small cell lung carcinoma; BC: breast cancer; OC: ovarian cancer; PCa: prostate cancer; LGG: low grade glioma; GBM: glioblastoma; PC: pancreatic cancer; AML: acute myeloid leukemia; CML: chronic myeloid leukemia; ALL: acute lymphocytic leukemia; CLL: chronic lymphocytic leukemia.
