# Supplementary material for: MiR-223-3p in Cancer Development and Cancer Drug Resistance: Same Coin, Different Faces
Source: Int J Mol Sci. 2024 Jul 26;25(15):8191. doi: 10.3390/ijms25158191 (PMC11311375; doi:10.3390/ijms25158191)
Supplement: Supplementary file 1 [file ijms-25-08191-s001.zip › Figure S2.pdf]

CRC	0	7
NSCLC	1	13
BC	0	1
OC	0	1
PCa	1	0
LGG / GBM	0	3
PC	0	2
AML	2	0
CML	1	0
ALL	0	1
CLL	0	0

Figure S2. Heatmap showing the number of articles cited in this review that defines miR-223 as decreased (blues) or increased (yellows) biomarker detectable in biological fluids or in EVs within them, for each type of cancer reviewed. The colour intensity of the heatmap is directly proportional to the number of references supporting the use of miR-223 as potential biomarker: the less is the number of references citing its decrease or increase the lighter is the blue or the yellow, respectively; the more is the the number of references citing its decrease or increase the deeper is the blue or the yellow. The number of references supporting the role of miR-223 is reported as arabic numbers within the heatmap, for each tumor type. CRC: colorectal carcinoma; NSCLC: non-small cell lung carcinoma; BC: breast cancer; OC: ovarian cancer; PCa: prostate cancer; LGG: low grade glioma; GBM: glioblastoma; PC: pancreatic cancer; AML: acute myeloid leukemia; CML: chronic myeloid leukemia; ALL: acute lymphocytic leukemia; CLL: chronic lymphocytic leukemia.
